# Identification of 50 K Illumina-chip SNPs associated with resistance to spot blotch in barley

**DOI:** 10.1186/s12870-017-1198-9

**Published:** 2017-12-28

**Authors:** Irina V. Bykova, Nina M. Lashina, Vadim M. Efimov, Olga S. Afanasenko, Elena K. Khlestkina

**Affiliations:** 1 Institute of Cytology and Genetics of the Siberian Branch of the Russian Academy of Sciences, Lavrentjeva ave. 10, Novosibirsk, 630090 Russia; 2 All-Russian Research Institute for Plant Protection, St. Petersburg, 196608 Russia; 3 Novosibirsk State University, Pirogova str., 1, Novosibirsk, 630090 Russia

**Keywords:** Association mapping, Barley, *Cochliobolus sativus*, GWAS, *Hordeum vulgare*, Resistance, SNP

## Abstract

**Background:**

Spot blotch, caused by *Cochliobolus sativus*, is one of the most widespread and harmful diseases in barley. Identification of genetic loci associated with resistance to *C. sativus* is of importance for future marker-assisted selection. The goal of the current study was to identify loci conferring seedling resistance to two different pathotypes of *C. sativus* in the Siberian spring barley core collection.

**Results:**

A total of 96 spring barley cultivars and lines were phenotyped at the seedling stage with two *C. sativus* isolates (Kr2 and Ch3). According to the Fetch-Steffenson rating scale 16%/17% of genotypes were resistant and 26%/30% were moderate-resistant to the Kr2/Ch3 isolates respectively. A total of 94 genotypes were analyzed with the barley 50 K Illumina Infinium iSELECT assay. From 44,040 SNPs, 40,703 were scorable, from which 39,140 were polymorphic. 27,319 SNPs passed filtering threshold and were used for association mapping. Data analysis by GLM revealed 48 and 41 SNPs for Kr2 and Ch3 isolates, respectively. After application of 5% Bonferroni multiple test correction, only 3 and 27 SNPs were identified, respectively. A total of three genomic regions were associated with the resistance. The region on chromosome 3H associated with Ch3-resistance was expanded between markers SCRI_RS_97417 and JHI-Hv50k-2016-158003 and included 11 SNPs, from which JHI-Hv50k-2016-157070, JHI-Hv50k-2016-156842 had the lowest *p*-values. These two SNPs were also significant in case of Kr2 isolate. The region on chromosome 2H included 16 loci (7 of them with the lowest *p*-values were tightly linked to BOPA2_12_11504). Three loci corresponding to this region had suggestive *p*-values in case of Kr2 tests, so the locus on chromosome 2H may also contribute to resistance to Kr2 isolate. The third region with significant *p*-value in case of Kr2 tests was identified on chromosome 1H at the locus JHI-Hv50k-2016-33568.

**Conclusions:**

Three genomic regions associated with the resistance to one or both isolates of *C. sativus* were identified via screening of the Siberian spring barley core collection. Comparison of their location with QTLs revealed previously either with biparental mapping populations studies or with GWAS of distinct germplasm and other isolates, demonstrated that resistance to isolates Kr2 and Ch3 is conferred by known spot blotch resistance loci. Information on SNPs related can be used further for development of DNA-markers convenient for diagnostics of resistance-associated alleles in barley breeding programs.

**Electronic supplementary material:**

The online version of this article (10.1186/s12870-017-1198-9) contains supplementary material, which is available to authorized users.

## Background

Spot blotch, caused by *Cochliobolus sativus*, is one of the most widespread and harmful diseases in barley. Identification of genetic loci associated with resistance to *C. sativus* is of importance for future development of diagnostic DNA-markers, which could be used for accelerated breeding of resistant cultivars. Two major approaches are usually applied for detection of genomic loci related with spot blotch resistance: (1) QTL-analysis of biparental mapping populations and (2) GWAS (genome-wide association studies; *syn.* – association mapping (AM)).

QTL-analysis of a total of 12 biparental mapping populations revealed loci for spot blotch resistance in all barley chromosomes [1–7]. Association mapping of spot blotch resistance also has been successfully used for identification of novel loci, exploiting different germplasms such as wild barley lines [8], US barley breeding germplasm [9], Virginia Tech winter barley lines [10], germplasm from Latin America [11], and USDA barley core collection [12]. The data accumulated from these studies on the one hand provide information on novel spot blotch resistance loci, on the other hand validate the AM approach, confirming results of studies of biparental populations. For example, in the earliest among these publications [8], 13 loci for *C. sativus* resistance were found on chromosomes 1H, 2H, 3H, 5H, and 7H, from which 7 loci were novel and 6 loci confirmed QTLs identified previously by analysis of biparental mapping populations. The latest among the mentioned AM studies [12] reported 10 chromosome regions associated with spot blotch, from which 6 were novel, suggesting effectiveness of investigating different barley germplasm and testing different *C. sativus* races for detection of new spot blotch resistance loci. AM approach has not been widely used for studying Russian barley germplasm, especially the Siberian barley collection.

The goal of the current study was to identify loci conferring seedling resistance to two different pathotypes of *C. sativus* (Kr2 and Ch3) in the Siberian spring barley core collection, using 50 K Illumina SNP-chip.

## Results

### Phenotyping

**Fig. 1:**
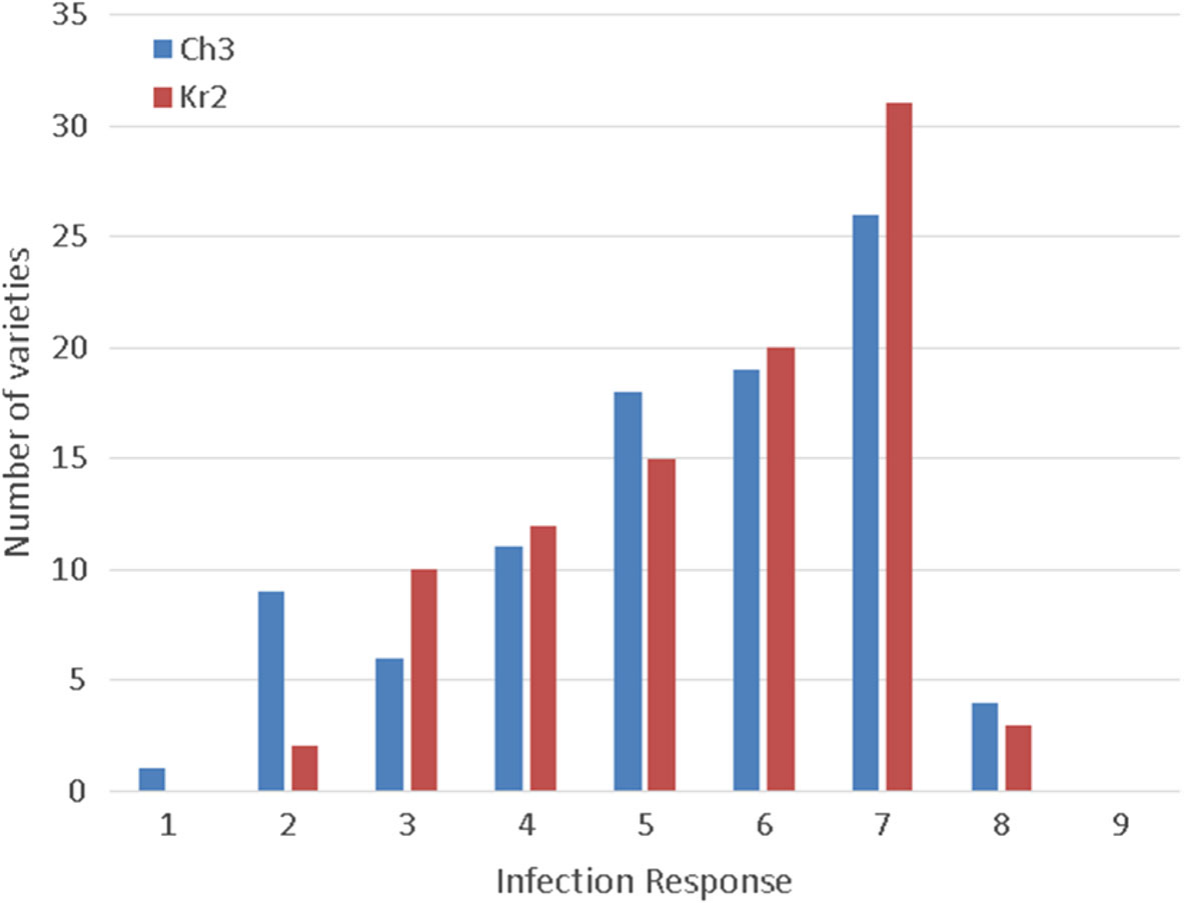
The frequency distributions for the average IRs to two *C. sativus* isolates of 93 barley genotypes

The IRs (infection responses) exhibited by barley genotypes were generally in agreement between three replicates within each experiment. The frequency distributions for the average IRs of barley genotypes are given in Fig. 1. Only three varieties (G-19980 and Biom in case of Kr2 tests and AC 0760258 for Ch3) showed heterozygosity and therefore were excluded from the further analysis. The majority (43.0%) of barley genotypes were susceptible to both *C. sativus* isolates; 53% - to Ch3 and 58% to Kr2 (IRs – 6-9). Nine varieties were resistant to both isolates: B-1, G-21219, Kolchan, Mutant 68, Omsky Golozyorny 2, Severny, Signal, Svetik, and Tanay. Sixteen varieties demonstrated resistance types of reaction (IRs – 1-3.9) to isolate Ch3 and 15 to Kr2. Moderate resistance (IRs – 4.0-5.9) to *C. sativus* isolate Ch3 was determined for 29 varieties, and to Kr2 for 24 varieties. Cultivars Emelya, Impuls, Kedr, Merit 57, and Reyd demonstrated isolate (race) specific resistance, they were susceptible to one isolate and resistant to another. Eleven varieties with moderate resistance (MR) to isolate Ch3 were susceptible to isolate Kr2, and vice versa 8 varieties with MR to isolate Kr2 were susceptible to Ch3 (Additional file 1).

### Genotyping and GWAS analysis

A total of 94 spring barley accessions were genotyped using the 50 K SNP iSelect array containing 44,040 SNPs, from which 40,703 were scorable. A total of 39,140 SNPs (89%) were polymorphic. The results of cluster analysis, showing the relationship between barley varieties are presented in Additional file 2. Cluster analysis revealed 4 major groups of accessions consisting of 38, 7, 19, 30 genotypes respectively.

After quality control filtering of the genotyping dataset, 27,319 SNPs (62,0%) were selected for GWAS. Data analysis by GLM allowed to detect 48 and 41 SNPs significantly associated with scores for Kr2 and Ch3 respectively (Tables 1 and 2, Additional file 3). After application of Bonferroni multiple test correction at 5% (*p* < 1.8302E-6), only 3 of 48 SNPs were significant for isolate Kr2 scores (Table 1), while for Ch3 27 of 41 had significant *p*-values (Table 2).

**Table 1:** SNPs associated with resistance to Kr2 isolate, revealed by GLM analysis and arranged according *p*-values

#	Marker	Chr	Physical map position (bp)	Genetic map position (cM)	*p*-value	Alleles	MAF
**1**	**JHI-Hv50k-2016-157070**	**3H**	**17,888,495**	**na**	6.04E-08*	**C/G**	**С(0.19)**
**2**	**JHI-Hv50k-2016-156842**	**3H**	**17,559,189**	**na**	1.20E-06*	**A/G**	**A(0.18)**
**3**	**JHI-Hv50k-2016-33568**	**1H**	**446,893,297**	**na**	1.29E-06*	**G/T**	**G(0.15)**
**4**	**JHI-Hv50k-2016-19966**	**1H**	**74,337,494**	**na**	**2.21E-06**	**G/T**	**G(0.27)**
**5**	**JHI-Hv50k-2016-407341**	**6H**	**462,119,913**	**na**	**6.35E-06**	**T/C**	**T(0.17)**
**6**	**SCRI_RS_83731**	**2H**	**na**	**57.0**	**9.86E-06**	**T/G**	**T(0.21)**
**7**	**SCRI_RS_162917**	**2H**	**517,229,083**	**57.2**	**9.86E-06**	**A/G**	**A(0.21)**
**8**	**SCRI_RS_233449**	**2H**	**520,779,376**	**57.0**	**9.86E-06**	**G/A**	**G(0.21)**
9	SCRI_RS_186769	2H	na	57.4	1.16E-05	G/C	G(0.22)
10	SCRI_RS_132839	2H	524,447,113	57.0	1.16E-05	A/G	A(0.22)
11	SCRI_RS_136740	2H	524,817,496	57.2	1.16E-05	A/C	A(0.22)
12	BOPA1_3355–605	2H	na	na	1.33E-05	C/A	C(0.19)
13	SCRI_RS_153880	2H	na	59.3	1.33E-05	T/C	T(0.19)
14	SCRI_RS_206529	2H	na	60.5	1.33E-05	A/G	A(0.19)
15	BOPA2_12_11504	2H	520,773,185	57.0	1.33E-05	A/G	A(0.19)
16	BOPA2_12_30108	2H	556,024,085	59.3	1.33E-05	A/C	A(0.19)
17	JHI-Hv50k-2016–98667	2H	559,662,446	na	1.33E-05	A/G	A(0.19)
18	SCRI_RS_141789	2H	551,217,066	59.3	1.33E-05	A/C	A(0.19)
19	SCRI_RS_182631	1H	74,327,682	46.8	1.44E-05	G/A	G(0.24)
20	SCRI_RS_188937	1H	74,325,931	46.8	1.44E-05	T/C	T(0.24)
21	JHI-Hv50k-2016-19943	1H	74,327,370	na	1.57E-05	C/T	C(0.26)
22	BOPA2_12_10159	1H	98,741,757	47.7	2.19E-05	C/A	C(0.33)
23	BOPA2_12_30438	1H	98,026,175	47.7	2.19E-05	A/G	A(0.33)
24	JHI-Hv50k-2016-20725	1H	96,478,890	na	2.19E-05	C/G	C(0.33)
25	BOPA2_12_10235	1H	80,265,474	47.7	2.42E-05	A/C	A(0.29)
26	JHI-Hv50k-2016-159556	3H	24,223,023	na	2.64E-05	G/A	G(0.41)
27	JHI-Hv50k-2016–99999	2H	589,523,785	na	3.04E-05	T/C	T(0.48)
28	JHI-Hv50k-2016-226122	4H	645,489	na	3.04E-05	A/G	A(0.37)
29	BOPA2_12_31179	1H	449,874,128	58.4	3.06E-05	C/G	C(0.13)
30	JHI-Hv50k-2016-20076	1H	80,292,944	na	3.41E-05	C/T	C(0.32)
31	SCRI_RS_85918	1H	80,292,373	47.7	3.41E-05	G/A	G(0.32)
32	JHI-Hv50k-2016–99440	2H	582,800,216	na	4.11E-05	A/G	A(0.36)
33	BOPA1_2634–2228	2H	520,264,176	na	4.53E-05	C/A	C(0.22)
34	BOPA1_5160–268	2H	520,778,105	na	4.53E-05	G/A	G(0.22)
35	SCRI_RS_191136	2H	520,437,064	57.0	4.53E-05	T/C	T(0.22)
36	JHI-Hv50k-2016-156387	3H	16,420,851	na	5.56E-05	C/A	C(0.10)
37	JHI-Hv50k-2016-156999	3H	17,817,242	na	5.80E-05	A/C	A(0.25)
38	JHI-Hv50k-2016-157182	3H	17,954,351	na	5.80E-05	T/A	T(0.25)
39	JHI-Hv50k-2016-155569	3H	15,256,329	na	5.85E-05	A/G	A(0.14)
40	BOPA2_12_10035	2H	463,231,068	56.7	6.51E-05	G/A	G(0.19)
41	SCRI_RS_161169	2H	483,288,774	56.7	6.51E-05	G/A	G(0.19)
42	JHI-Hv50k-2016–92202	2H	309,655,073	na	2.17E-04	T/C	T(0.21)
43	BOPA2_12_30179	2H	na	56.4	2.94E-04	A/G	A(0.19)
44	SCRI_RS_97417	3H	15,255,540	12.1	3.91E-04	C/T	C(0.34)
45	JHI-Hv50k-2016-156336	3H	16,375,848	na	4.23E-04	A/T	A(0.11)
46	BOPA1_5254–1845	2H	175,163,708	na	4.59E-04	G/A	G(0.18)
47	SCRI_RS_109192	2H	175,053,470	na	4.59E-04	G/T	G(0.18)
48	JHI-Hv50k-2016-155951	3H	15,469,647	na	7.68E-04	T/A	T(0.30)

1–3 (underlined bold): SNPs, which are significant according Bonferroni multiple test correction at 5% (*p* < 1.8302E-6).4–8 (bold): suggestive SNPs. MAF – Minor allele frequency. Chr – chromosome, na – not available. Genetic map positions are given according Morex / Barke iSelect map (http://bioinf.hutton.ac.uk/iselect/app/)

**Table 2:** SNPs associated with resistance to Ch3 isolate, revealed by GLM analysis and arranged according *p*-values

#	Marker	Chr	Physical map position (bp)	Genetical map position (cM)	*p*-value	Alleles	MAF, %
**1**	**JHI-Hv50k-2016-157070**	**3H**	**17,888,495**	**na**	6.04E-08*	**C/G**	**C(0.19)**
**2**	**JHI-Hv50k-2016-156842**	**3H**	**17,559,189**	**na**	1.20E-06*	**A/G**	**A(0.18)**
**3**	**JHI-Hv50k-2016-156999**	**3H**	**17,817,242**	**na**	5.71E-11*	**A/C**	**A(0.25)**
**4**	**JHI-Hv50k-2016-156329**	**3H**	**16,375,436**	**na**	3.26E-09*	**G/A**	**G(0.25)**
**5**	**JHI-Hv50k-2016-157182**	**3H**	**17,954,351**	**na**	3.67E-09*	**T/A**	**T(0.25)**
**6**	**JHI-Hv50k-2016-156387**	**3H**	**16,420,851**	**na**	1.45E-08*	**C/A**	**C(0.10)**
**7**	**JHI-Hv50k-2016-156336**	**3H**	**16,375,848**	**na**	1.05E-07*	**A/T**	**A(0.11)**
**8**	**BOPA1_3355–605**	**2H**	**na**	**na**	1.19E-07*	**C/A**	**C(0.19)**
**9**	**SCRI_RS_153880**	**2H**	**na**	**59.3**	1.19E-07*	**T/C**	**T(0.19)**
**10**	**SCRI_RS_206529**	**2H**	**na**	**60.5**	1.19E-07*	**A/G**	**A(0.19)**
**11**	**BOPA2_12_11504**	**2H**	**520,773,185**	**57.0**	1.19E-07*	**A/G**	**A(0.19)**
**12**	**BOPA2_12_30108**	**2H**	**556,024,085**	**59.3**	1.19E-07*	**A/C**	**A(0.19)**
**13**	**JHI-Hv50k-2016–98667**	**2H**	**559,662,446**	**na**	1.19E-07*	**A/G**	**A(0.19)**
**14**	**SCRI_RS_141789**	**2H**	**551,217,066**	**59.3**	1.19E-07*	**A/C**	**A(0.19)**
**15**	**JHI-Hv50k-2016-156833**	**3H**	**17,558,292**	**na**	1.73E-07*	**T/A**	**T(0.29)**
**16**	**SCRI_RS_83731**	**2H**	**na**	**57.0**	1.84E-07*	**T/G**	**T(0.21)**
**17**	**SCRI_RS_162917**	**2H**	**517,229,083**	**57.2**	1.84E-07*	**A/G**	**A(0.21)**
**18**	**SCRI_RS_233449**	**2H**	**520,779,376**	**57.0**	1.84E-07*	**G/A**	**G(0.21)**
**19**	**JHI-Hv50k-2016-155569**	**3H**	**15,256,329**	**na**	4.30E-07*	**A/G**	**A(0.14)**
**20**	**JHI-Hv50k-2016-158003**	**3H**	**18,788,405**	**na**	5.36E-07*	**G/A**	**G(0.27)**
**21**	**SCRI_RS_97417**	**3H**	**15,255,540**	**12.1**	1.35E-06*	**C/T**	**C(0.34)**
**22**	**SCRI_RS_186769**	**2H**	**na**	**57.4**	1.35E-06*	**G/C**	**G(0.22)**
**23**	**BOPA1_2634–2228**	**2H**	**520,264,176**	**na**	1.35E-06*	**C/A**	**C(0.22)**
**24**	**BOPA1_5160–268**	**2H**	**520,778,105**	**na**	1.35E-06*	**G/A**	**G(0.22)**
**25**	**SCRI_RS_132839**	**2H**	**524,447,113**	**57.0**	1.35E-06*	**A/G**	**A(0.22)**
**26**	**SCRI_RS_136740**	**2H**	**524,817,496**	**57.2**	1.35E-06*	**A/C**	**A(0.22)**
**27**	**SCRI_RS_191136**	**2H**	**520,437,064**	**57.0**	1.35E-06*	**T/C**	**T(0.22)**
**28**	**BOPA2_12_30179**	**2H**	**na**	**56.4**	**2.15E-06**	**A/G**	**A(0.19)**
**29**	**JHI-Hv50k-2016–92202**	**2H**	**309,655,073**	**na**	**2.96E-06**	**T/C**	**T(0.21)**
**30**	**BOPA2_12_10035**	**2H**	**463,231,068**	**56.7**	**3.10E-06**	**G/A**	**G(0.19)**
**31**	**SCRI_RS_161169**	**2H**	**483,288,774**	**56.7**	**3.10E-06**	**G/A**	**G(0.19)**
**32**	**BOPA1_5254–1845**	**2H**	**175,163,708**	**na**	**5.42E-06**	**G/A**	**G(0.18)**
**33**	**SCRI_RS_109192**	**2H**	**175,053,470**	**na**	**5.42E-06**	**G/T**	**G(0.18)**
**34**	**JHI-Hv50k-2016-226122**	**4H**	**645,489**	**na**	**6.20E-06**	**A/G**	**A(0.37)**
**35**	**JHI-Hv50k-2016-155951**	**3H**	**15,469,647**	**na**	**8.49E-06**	**T/A**	**T(0.30)**
**36**	**JHI-Hv50k-2016-153016**	**3H**	**7,847,727**	**na**	**9.22E-06**	**A/G**	**G(0.10)**
**37**	**JHI-Hv50k-2016-445854**	**7H**	**10,393,349**	**na**	**9.28E-06**	**A/G**	**G(0.48)**
**38**	**JHI-Hv50k-2016-17526**	**1H**	**32,102,667**	**na**	**9.88E-06**	**G/A**	**A(0.42)**
**39**	**SCRI_RS_162708**	**7H**	**56,674,116**	**47.9**	**9.88E-06**	**A/G**	**G(0.29)**
40	JHI-Hv50k-2016-159556	3H	24,223,023	na	1.60E-04	G/A	G(0.41)
41	BOPA2_12_31179	1H	449,874,128	58.4	3.54E-04	C/G	C(0.13)

1–27 (underlined bold): SNPs, which are significant according Bonferroni multiple test correction at 5% (*p* < 1.8302E-6).28–39 (bold): suggestive SNPs. MAF – Minor allele frequency. Chr – chromosome, na – not available. Genetic map positions are given according Morex / Barke iSelect map (http://bioinf.hutton.ac.uk/iselect/app/)

**Fig. 2:**
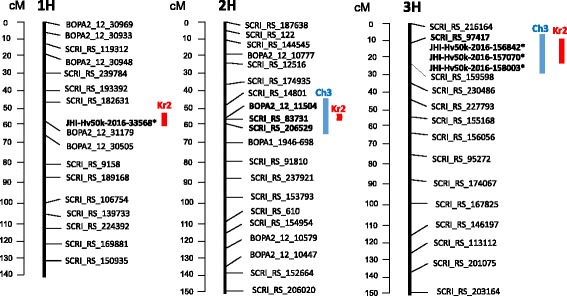
Location of genome fragments associated with resistance to spot blotch isolates Kr (red) and Ch3 (blue) using Morex / Barke iSelect map (http://bioinf.hutton.ac.uk/iselect/app/). Dashed line - suggestive SNP. * - markers are placed according physical position relative to genetically mapped SNPs

A total of three genomic regions were associated with resistance. The region on chromosome 3H associated with Ch3-resistance was expanded between markers SCRI_RS_97417 and JHI-Hv50k-2016-158003 and included 11 SNPs, from which JHI-Hv50k-2016-157070, JHI-Hv50k-2016-156842 had the lowest *p*-values (Table 2). These two SNPs were also significantly associated with resistance to Kr2 isolate (Table 1). The region on chromosome 2H included 16 loci (Table 2), 7 of them with the lowest *p*-values were tightly linked to BOPA2_12_11504. Three loci corresponding to this region had suggestive *p*-values in case of Kr2 tests (Table 1)), so the locus on chromosome 2H may also contribute to resistance to Kr2-isolate. The third region with significant *p*-value was identified on chromosome 1H at the locus JHI-Hv50k-2016-33568, it was associated with Kr2-resistance (Table 1). The genome regions identified are visualized at Fig. 2 using the iSelect linkage maps.

Analysis of SNPs with lowest *p*-values as marker loci to be converted into the convenient diagnostic markers showed that some of the SNPs have a potential for identification of resistant varieties with high accuracy. For example, 14 of 19 (74%) varieties resistant to one or both isolates are characterised by C-allelic variant at the JHI-Hv50k-2016-157070 locus. This allele also detected 4 additional varieties having medium-resistance to one or both isolates but was absent in genotypes sensitive to both isolates.

## Discussion

From 94 accessions genotyped 47 (50%) were cultivars and lines developed in breeding centers located in Siberia. Other 47 accessions were cultivars and lines maintained in the Siberian spring barley collection, but originating from other regions and countries. Cluster analysis revealed 4 major groups of accessions (Additional file 2). Two big groups consisting of 38 (group I) and 30 (group IV) genotypes noticeably differed by percentage of Siberian cultivars and lines (73% and 17% respectively). Groups II and III were “half-Siberian” (Additional file 2). Group I had the highest percentage of varieties resistant to both isolates of *C. sativus* (18% vs 0, 3 and 5% in other three groups) and the lowest percentage of susceptible varieties (32% vs 47, 48 and 50% in other groups) to both isolates (Additional file 2). This may suggest essential contribution of Siberian varieties to resistant germplasm of spring barleys studied. Indeed, we noticed that among accessions resistant to both isolates 67% had Siberian origin.

GWAS revealed three genomic regions (on chromosomes 1H, 2H and 3H) associated with resistance to one or both isolates (Tables 1 and 2, Fig. 2). In spite of small sample size analyzed in the current study, we did not reveal false positive loci. Comparison of three genomic regions identified in our study with locations of *C. sativus* resistance QTLs revealed previously, showed that each of the three regions contains known spot blotch resistance loci.

### Chromosome 1H

SNP associated with resistance to *C. sativus* isolate Kr2 was revealed between 50 and 60 cM of Chromosome 1H genetic map. The presence of QTL for spot blotch resistance in barley chromosome 1H within 50 and 60 cM was reported in several studies. In 1996, Steffenson et al. [1] reported QTL for adult resistance to *C. sativus* on chromosome 1H between markers ABG500–ABG494 (53.6–61.2 cM, according consensus map [13]). In 2005, Bilgic et al. [2] has found major locus for seedling resistance in the same region using Steptoe / Morex (R) mapping population. In 2010, Roy et al. [8] has revealed seedling resistance locus at 59,7 cM by association mapping.

Later, spot blotch resistance locus was found in barley chromosome 1H within 40 and 50 cM. Association with this region was reported in 2013 by Gutierrez et al. [11], who used AM-approach exploiting DArT-markers. At the same time, Zhou and Steffenson [9] have found locus for both adult and seedling resistance (associated with BOPA SNP markers 11_10764, 11_10275 and 12_30336) at 41–43 cM of chromosome 1H. In 2015, Afanasenko et al. [7] has revealed QTL for seedling resistance (at the SNP-locus BOPA11_10764, *syn.* BOPA1_5381–1950; position in iSelect map - 41.5 cM) by analysis of biparental mapping population Zernogradsky 85 (R) / Ranny 1. In 2016, Haas et al. [6] has found QTL for seedling resistance (at the SNP-locus BOPA2_12_30404; position in iSelect map - 48.1 cM) by analysis of biparental population PI 466423 (R) / Rasmusson. Recently, Wang et al. [12] reported about association of the region 42–44 cM (markers SCRI_RS_193392, SCRI_RS_153785, SCRI_RS_170878, SCRI_RS_170869, SCRI_RS_189483, BOPA1_5381_1950) with seedling resistance.

The resistance QTL found on chromosome 1H in our study is likely coincident with previously identified QTLs in the interval 40–60 cM [1, 2, 6–9, 11, 12]. Besides chromosome region within 40–60 cM, two more regions of chromosome 1H are associated with resistance to spot blotch: distal part of the long arm (1HL; QTL at the SNP-locus BOPA_11_10433, *syn.* BOPA1_3201–603; position in iSelect map - 87.0 cM [7]) and the short arm (1HS; gene *Rcs6* at ca. 15 cM [3, 14] and QTL at the DArT-locus bPb–9604; 16.9 cM [5]).

### Chromosome 2H

The region on chromosome 2H (within 57–60 cM) revealed in our study for Ch3 isolate resistance included 16 SNP loci (Table 2). Three SNP loci corresponding to this region had suggestive *p*-values in case of Kr2 tests (Table 1), so the resistance locus on chromosome 2H may also contribute to resistance to Kr2-isolate (Fig. 2).

The resistance QTL found on chromosome 2H in our study is likely coincident with previously identified QTLs for seedling resistance [2, 7]. In 2005, Bilgic et al. [2] detected QTL in the interval 27.1–46.9 cM (markers Rbcs–ABG459) using Steptoe / Morex (R) mapping population. Later, Afanasenko et al. [7] revealed QTL at the SNP-locus BOPA_11_11015 (*syn.* BOPA1_946–2500; position in iSelect map - 54.2 cM) by analysis of biparental mapping population Zernogradsky 85 (R) / Ranny 1.

Besides this chromosome region another part of chromosome 2H (distal part of the long arm) is associated with resistance to spot blotch. Wang et al. [12] revealed significant association with SCRI_RS_152664 (138.6 cM).

### Chromosome 3H

The region of chromosome 3H associated with Ch3-resistance was expanded between markers SCRI_RS_97417 and JHI-Hv50k-2016-158003 and included 11 SNPs, from which JHI-Hv50k-2016-157070, JHI-Hv50k-2016-156842 had the lowest *p*-values (Table 2). These two SNPs were also significant with resistance to Kr2 isolate (Table 1). Position of genetically mapped marker from this set is 12.1 cM.

The resistance QTL found on chromosome 3H in our study is likely coincident with some of the previously identified QTLs. Bovill et al. [4] revealed QTLs for adult plant resistance to spot blotch within 2–28 cM of chromosome 3H, using different mapping populations. Later, Zhou and Steffenson [9] revealed seedling and adult resistance loci on chromosome 3H in positions 9.6 cM (marker BOPA_12_30818) and 19.2 cM (BOPA_11_20742, BOPA_11_10565), exploiting association mapping approach.

More proximal loci on chromosome 3H also have been found. Bilgic et al. (2005) revealed locus for seedling resistance in the interval 28.7–42.4 (markers ABC171-MWG584) using Steptoe/Morex (R) mapping population. Grewal et al. [5] revealed seedling resistance QTL in the interval 24.9–31.1 cM (marker bPb-3565), and 2 adult plant resistance QTLs in the intervals 23.0–24.9 cM (marker bPb-6127) and 31.8–43.3 cM (marker E40M61.1), using DH recombinant lines from the cross CDC Bold / TR251 (R). Wang et al. [12] reported two regions on chromosome 3H, each associated with different isolates: BOPA1_3906_558 at 25.3 cM and BOPA1_5960_1302 at 66.2 cM.

## Conclusions

Three genomic regions associated with the resistance to one or both isolates of *C. sativus* were identified via screening of the Siberian spring barley core collection. Comparison of their location with QTLs revealed previously either with biparental mapping populations studies or with GWAS of distinct germplasm and other isolates, demonstrated that resistance to isolates Kr2 and Ch3 is conferred by known spot blotch resistance loci. Information on SNPs related with seedling resistance to Kr2 and Ch3 isolates as well as results of variety resistance assessment can be useful for further marker-assisted selection of spring barley. SNPs could be converted into convenient diagnostic markers (for example CAPs). Varieties B-1, G-21219, Kolchan, Mutant 68, Omsky Golozyorny 2, Severny, Signal, Svetik, and Tanay can be recommended as donors of stable resistance to spot blotch.

## Methods

### Plant materials

A total of 96 accessions from the Siberian spring barley core collection were selected for phenotyping. Half of these accessions were cultivars and lines developed in breeding centers located in Siberia (from Altay, Buryatiya, Irkutsk, Kemerovo, Novosibirsk, Omsk, Tomsk, Tyumen and Yakutiya regions). Another half of accessions were cultivars and lines maintained in the Siberian spring barley collection, but originating from other regions of the Russian Federation (Arkhangelsk, Chelyabinsk, Dagestan, Kirov, Krasnodar, Leningrad, Moscow, Orenburg, Primorsky, Rostov, Samara, Stavropol, Sverdlovsk, and Voronezh regions) or other countries (Ethiopia, Germany, Kazakhstan, Kyrgyzstan, Sweden, Switzerland, Ukraine, USA, Yemen). Plants were grown in plastic trays (12 × 17 cm and 7 cm depth) filled with soil “Terra vita” (standard fertilized (15–27-30, NPK) mixture of peat and soil for seedlings) in the climatic room at 20-22 °C with alternating 16 h periods of light/8 h of for darkness (exposure 5000 lx).Two plants of each barley genotype and cultivar Harrington as a susceptible control were evaluated for resistance in each tray in a completely randomized design with three replicates.

### Pathogen isolates and culture conditions

Two *C. sativus* isolates of different origin were used for seedling resistance evaluation: Ch3 (North West of Russia, Leningrad region) and Kr2 (South of European part of Russia, Krasnodar region). Isolate Ch3 has been used in previous studies for evaluation of resistance of a wide set of barley genotypes and showed high aggressiveness on susceptible barley genotypes. Isolate Kr2 was chosen because of its origin and good sporulation ability. These isolates were derived from single conidia in 2015 and were stored in glass tubes at 4 °C on the CLM medium (modified Czapek medium with lactose, and urea) containing (g per 1 l): 0.5 KH2PO4, 0.5 MgSO4, 0.5 KCl, 1.2 urea, 20 lactose, and 20 agar.

Isolates were sub-cultured on the same medium at 20-22 °C with a 12 h photoperiod. After 10–12 days the culture were flooded with distilled water and conidia were dislodged with a sterile spatula and filtering through two layers of cheesecloth. The conidia concentration was determined by haemocytometer and was adjusted to 10,000 conidia/ml.

Plants were inoculated by spraying with conidia suspension 12–14 days after planting (two-three leaf stage). Approximately 10 ml per one tray (12 barley accessions) was used for inoculation. Inoculated plants were covered with plastic bags and incubated at 20-22 °C for 24 h in darkness at 100% relative humidity and then grown at the same temperature with 16/8 h light/dark photoperiod at 70% RH.

### Assessment of infection response

Infection responses (IRs) were recorded 10 days post-inoculation at the 2- to 3-leaf stage using the one to nine rating scale of Fetch and Steffenson [15]. This scale is based on the lesion size and the degree of associated chlorosis. Low IRs 1.0–3.9 (minute to small necrotic lesions with no or very slight diffuse marginal chlorosis) corresponds to resistance (R), 4.0–5.9 (medium-sized necrotic lesions with a distinct but restricted chlorotic margin) – to moderate resistance (MR), and high IRs – 6.0-9.0 (large necrotic lesions with distinct chlorotic margins and varying degrees of expanding diffuse chlorosis) – to susceptibility (S) [15]. Types of reactions were recorded only if susceptible cultivar Harrington demonstrated high IRs.

### DNA genotyping and data analysis

DNA was extracted from seedlings of individual plants using the DNeasy Plant Mini Kit (Qiagen, CA, USA). All samples were genotyped using the barley 50,000 Illumina iSelect SNP array at the Traitgenetics GmbH (Gatersleben, Germany).

Phylogenetic analysis was conducted using MEGA software v6.0 [16]. Genetic relationships were calculated by Maximum Likelihood (ML) analysis, Tamura-Nei model. Number of bootstrap replications was 500.

The SNP dataset was filtered using Excel software. Markers with minor allele frequency ≥ 0.10 were considered for GWAS. Markers with missing data were deleted. GWAS was performed using GLM (generalized linear model) with the help of TASSEL 5 package [17], based on phenotypic (scores for resistance for 2 *C. sativus* isolates) and genotypic (27,319 informative SNPs) data for the set of 94 barley accessions. Post association analysis corrections were performed using Bonferroni multiple test. Additional information on SNPs was extracted from resources BARLEYMAP (http://floresta.eead.csic.es/barleymap [18]) and iSelect (http://bioinf.hutton.ac.uk/iselect/app/).

## Additional files

Additional file 1: Table with results of spot blotch resistance assessment within the Siberian spring barley core collection. R – resistant (1.0–3.9); MR moderate resistant – (4.0–5.9); S – susceptible (6.0–9.0); “-” – failed. (DOCX 19 kb)

Additional file 2: Relationships between barley varieties based on genotyping with 50 K SNP iSelect array. I, II, III and IV – main clusters. Varieties names: black – susceptible to one *C. sativus* isolate and resistant (moderate resistant) to another; green – moderate resistant to one isolate and resistant/ moderate resistant to another; green underlined – resistant to both isolates; red – susceptible for both isolates. Diagrams corresponding to the clusters: orange/blue – non-Siberian/Siberian varieties; green/red/grey – resistant to both isolates/ susceptible to both isolates / others. (PDF 197 kb)

Additional file 3: Manhattan plots of the association mapping study for barley resistance to spot blotch isolates Kr2 (A), Ch3 (B) and quantile-quantile (QQ) plots of GWAS for Kr2 (C) and Ch3 (D). (PDF 307 kb)
