# Supplementary material for: Identification of 50 K Illumina-chip SNPs associated with resistance to spot blotch in barley
Source: BMC Plant Biol. 2017 Dec 28;17(Suppl 2):250. doi: 10.1186/s12870-017-1198-9 (PMC5751810; doi:10.1186/s12870-017-1198-9)
Supplement: Supplementary file 1 — Table with results of spot blotch resistance assessment within the Siberian spring barley core collection. R – resistant (1.0–3.9); MR moderate resistant – (4.0–5.9); S – susceptible (6.0–9.0); “-” – failed. (DOCX 19 kb) [file 12870_2017_1198_MOESM1_ESM.docx]

**Additional file 1:** Table with results of spot blotch resistance assessment within the Siberian spring barley core collection. R – resistant (1.0 – 3.9); MR moderately resistant – (4.0-5.9); S – susceptible (6.0-9.0); “-” – failed.

| **Cultivar/line name** | **Reaction to Kr2** | | **Reaction to Ch3** | |
| --- | --- | --- | --- | --- |
|  | **Value** | **Rating scale** | **Value** | **Rating scale** |
| Abyssinia | **7** | S | **7** | S |
| AC 0760258 | **7,5** | S |  | - |
| Acha | **6** | S | **4,7** | MR |
| Agul | **7** | S | **6,5** | S |
| Alag-Erdene | **7** | S | **7** | S |
| Aley | **4** | MR | **2,3** | R |
| Altan-Bulag | **8** | S | **8** | S |
| Alyn-Buya | **7** | S | **7** | S |
| Anna | **7** | S | **7** | S |
| Archekas | **6** | S | **6** | S |
| Arna | **6** | S | **7** | S |
| Avalon | **6,7** | S | **5,7** | MR |
| B-1 | **3** | R | **2** | R |
| Bagan | **4(5)** | MR | **7** | S |
| Barkhatny | **4** | MR | **4,3** | MR |
| Belogorsky | **7,5** | S | **7,7** | S |
| Bezenchuksky 2 | **4,5** | MR | **6,3** | S |
| Biom | **-** | - | **2,7** | R |
| Brachny | **5** | MR | **5,3** | MR |
| Chelyabinsky 70 | **5** | MR | **5** | MR |
| Dobry | **6,3** | S | **6,7** | S |
| Donetsky 8 | **6,7** | S | **7** | S |
| Emelya | **7** | S | **3,7** | R |
| G-19951 | **6** | S | **5** | MR |
| G-19980 | **-** | - | **3,3** | R |
| G-21038 | **5** | MR | **4,5** | MR |
| G-21219 | **3,3** | R | **2** | R |
| G-21671 | **3** | R | **4,5** | MR |
| G-21672 | **5,3** | MR | **5** | MR |
| Golozyorny 1 | **7** | S | **8** | S |
| Granal | **5,5** | MR | **7** | S |
| Ilmen | **6,7** | S | **6,7** | S |
| Impuls | **3,7** | R | **7** | S |
| Jngve | **7** | S | **6** | S |
| Kedr | **3,5** | R | **6,3** | S |
| Kolchan | **3,5** | R | **2** | R |
| Krasnoyarsky 1 | **8** | S | **7** | S |
| Krasnoyarsky 91 | **5** | MR | **4** | MR |
| Krymchak 55 | **4(5)** | MR | **5** | MR |
| Kuryer | **7** | S | **7** | S |
| L-1 | **7** | S | **7,3** | S |
| L-1285 | **6,5** | S | **7** | S |
| L-259/528 | **7** | S | **7** | S |
| L-421 | **5** | MR | **6** | S |
| Manych 459 | **4,3** | MR | **5** | MR |
| Mayak | **6,5** | S | **6,5** | S |
| Medikum | **5** | MR | **7** | S |
| Melius | **7** | S | **6** | S |
| Merit 57 | **7,7** | S | **3** | R |
| Mestny Dagestanian | **7** | S | **8** | S |
| Mestny Ethiopian | **7** | S | **7** | S |
| Mestny Primorsky | **4** | R | **5,3** | MR |
| Mestny Yakutian | **5,3** | MR | **4,7** | MR |
| Moskovsky 121 | **7,3** | S | **7** | S |
| Mutant 68 | **3,7** | R | **3** | R |
| Narymchanin | **5** | MR | **4** | MR |
| NGB 112412 | **7** | S | **8** | S |
| Nikita | **6** | S | **6** | S |
| Nosovsky 11 | **7,7** | S | **7,3** | S |
| Novosibirsky 80 | **4,3** | MR | **5** | MR |
| Nutans 274 | **6,7** | S | **6,5** | S |
| Nutans 970 | **7** | S | **7** | S |
| Obskoy | **7** | S | **7** | S |
| Omsky 13709 | **4,7** | MR | **7** | S |
| Omsky 85 | **5,3** | MR | **6** | S |
| Omsky golozyorny 1 | **6,3** | S | **6,5** | S |
| Omsky golozyorny 2 | **2,3** | R | **2,3** | R |
| Orenburgsky kormovoy | **7** | S | **6,5** | S |
| Oskar | **7** | S | **6** | S |
| Pallidum 394 | **7,3** | S | **7** | S |
| Priekulsky 14 | **5,5** | MR | **6** | S |
| Reyd | **7** | S | **3,3** | R |
| Sasha | **7** | S | **5,3** | MR |
| Selection from Tyal | **7** | S | **7,3** | S |
| Severny | **3** | R | **2,7** | R |
| Signal | **4** | R | **2** | R |
| Simvol | **3** | R | **5** | MR |
| Slavyansky | **7** | S | **5,7** | MR |
| Sobolyok | **6,3** | S | **6,5** | S |
| Svetik | **4** | R | **1** | R |
| Symbat | **6,7** | S | **7** | S |
| Taganay | **6** | S | **5** | MR |
| Talan | **7** | S | **4,3** | MR |
| Tanay | **3(4)** | R | **2,7** | R |
| Tarsky 1 | **4,7** | MR | **5,3** | MR |
| Tatum | **8** | S | **4,3** | MR |
| Temp | **6,5** | S | **6,5** | S |
| Vikont | **7** | S | **5,7** | MR |
| Viner | **5** | MR | **5** | MR |
| Viner mutant | **2,7** | R | **5** | MR |
| Vorsinsky 2 | **5,5** | MR | **3,7** | R |
| Vybor | **6** | S | **6** | S |
| Wial | **6,7** | S | **5,3** | MR |
| Zalarinets | **5** | MR | **4** | MR |
| Zernogradsky 86 | **7** | S | **7,3** | S |
| Zolotnik | **6** | S | **4** | MR |
| **Harrington** | **7,5** | S | **8** | S |
| **(control)** |  |  |  |  |
