# Supplementary figures and images for: Identification of 50 K Illumina-chip SNPs associated with resistance to spot blotch in barley
Source: BMC Plant Biol. 2017 Dec 28;17(Suppl 2):250. doi: 10.1186/s12870-017-1198-9 (PMC5751810; doi:10.1186/s12870-017-1198-9)

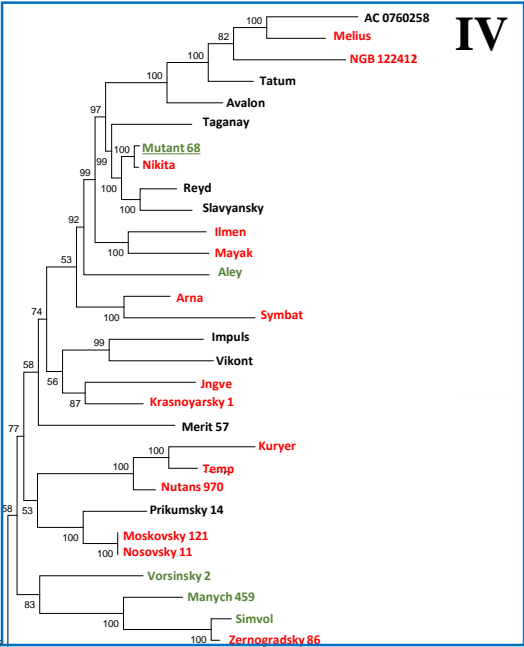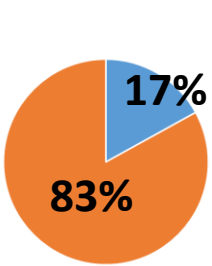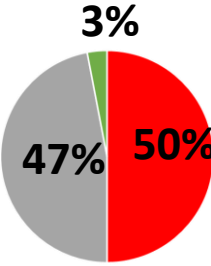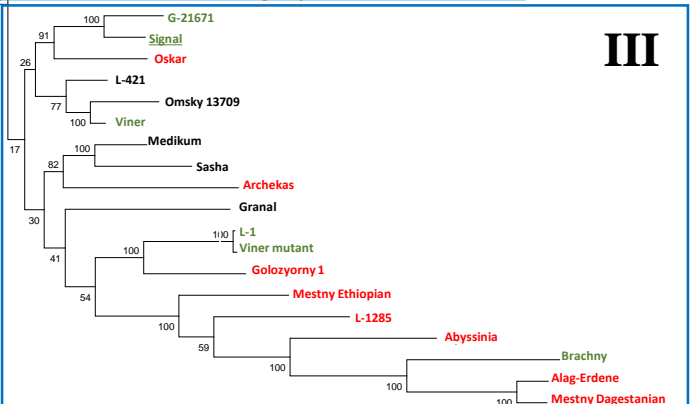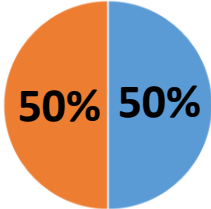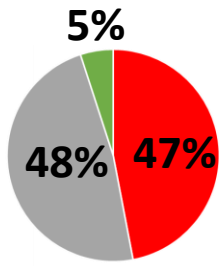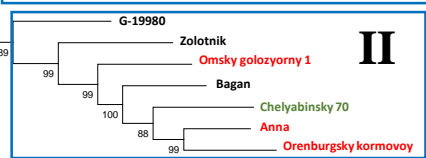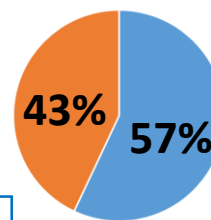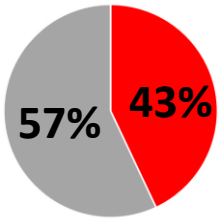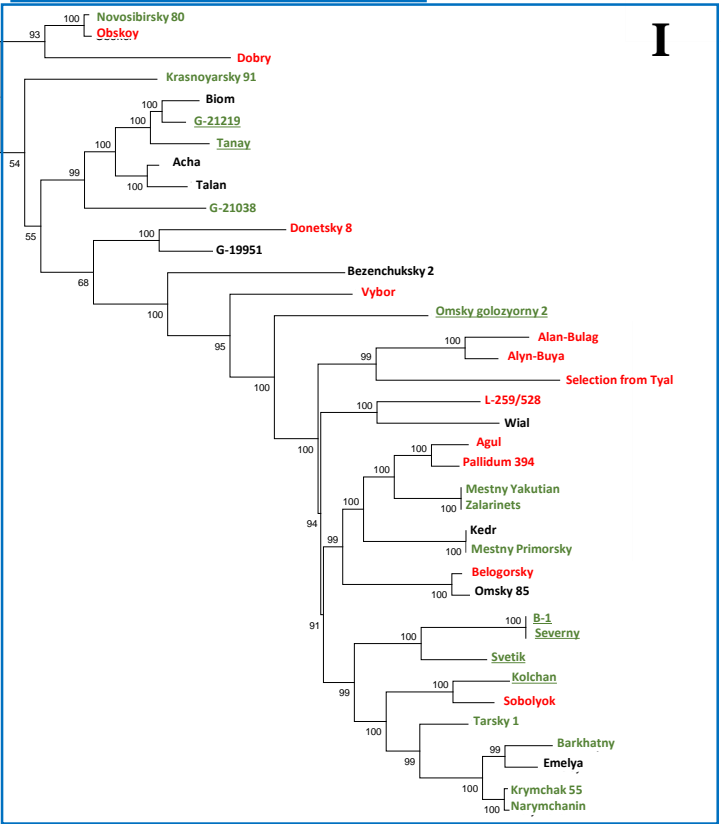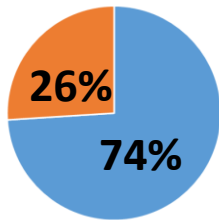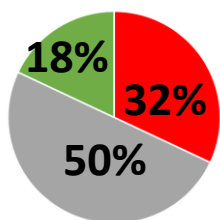

0.05

Supplement: Supplementary file 2 — Relationships between barley varieties based on genotyping with 50 K SNP iSelect array. I, II, III and IV – main clusters. Varieties names: black – susceptible to one C. sativus isolate and resistant (moderate resistant) to another; green – moderate resistant to one isolate and resistant/ moderate resistant to another; green underlined – resistant to both isolates; red – susceptible for both isolates. Diagrams corresponding to the clusters: orange/blue – non-Siberian/Siberian varieties; green/red/grey – resistant to both isolates/ susceptible to both isolates / others. (PDF 197 kb) [file 12870_2017_1198_MOESM2_ESM.pdf]
