# Supplementary material for: Identification of 50 K Illumina-chip SNPs associated with resistance to spot blotch in barley
Source: BMC Plant Biol. 2017 Dec 28;17(Suppl 2):250. doi: 10.1186/s12870-017-1198-9 (PMC5751810; doi:10.1186/s12870-017-1198-9)
Supplement: Supplementary file 3 — Manhattan plots of the association mapping study for barley resistance to spot blotch isolates Kr2 (A), Ch3 (B) and quantile-quantile (QQ) plots of GWAS for Kr2 (C) and Ch3 (D). (PDF 307 kb) [file 12870_2017_1198_MOESM3_ESM.pdf]

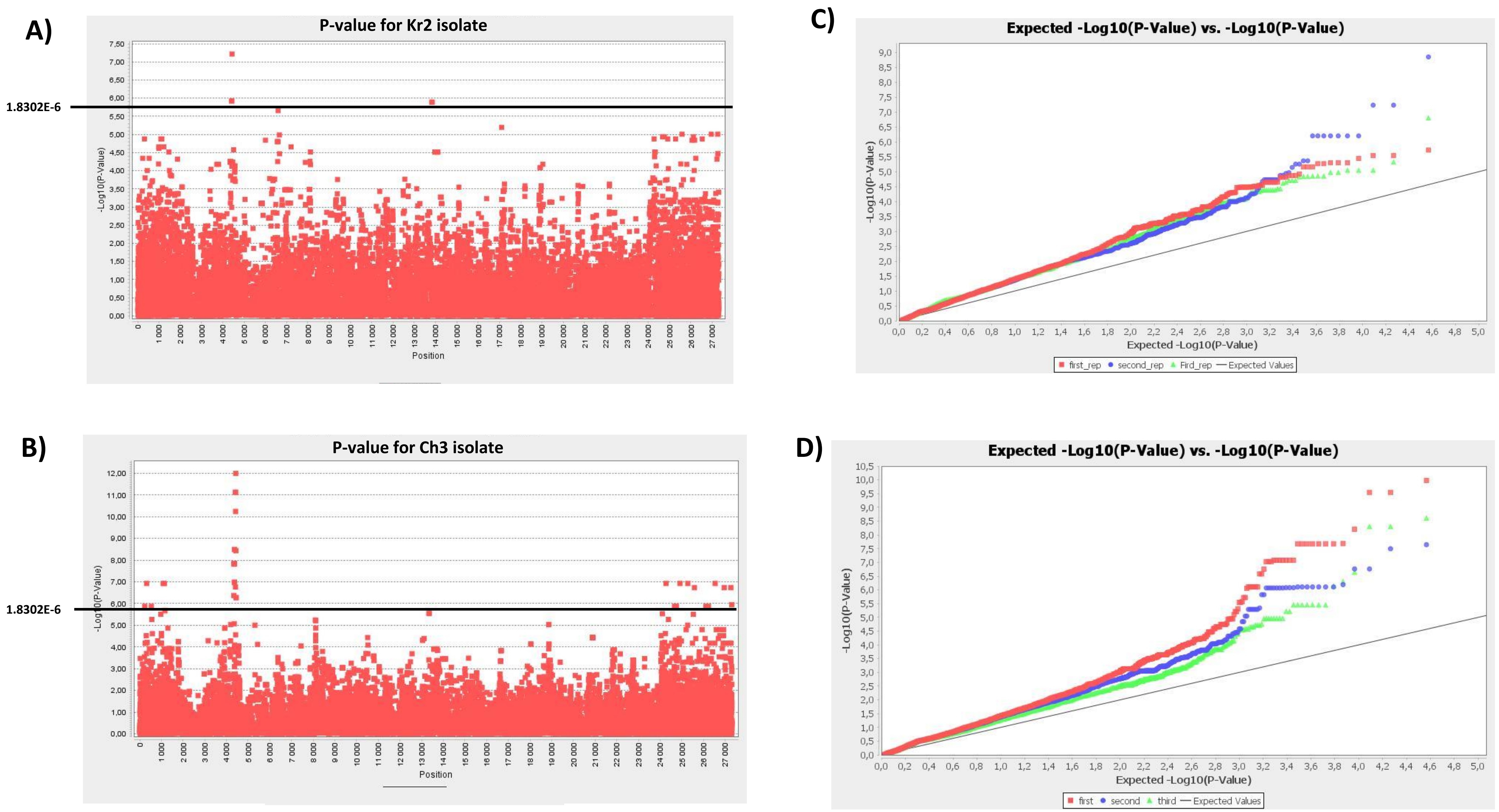

A)
P-value for Kr2 isolate
1.8302E-6
-Log10(P-Value)
Position
B)
P-value for Ch3 isolate
1.8302E-6
-Log10(P-Value)
Position
C)
Expected -Log10(P-Value) vs. -Log10(P-Value)
-Log10(P-Value)
Expected -Log10(P-Value)
first_rep
second_rep
Fird_rep
Expected Values
D)
Expected -Log10(P-Value) vs. -Log10(P-Value)
-Log10(P-Value)
Expected -Log10(P-Value)
first
second
third
Expected Values
